# The Week's Work

**Published:** 1910-12-31

**Authors:** 


### The Week's Work.

### THE WEST LONDON HOSPITAL APPOINTMENT.

On page 412 we publish, under the heading "An Official Disclaimer," a letter from the medical staff of the West London Hospital with reference to a note published in "Week's Work" of December 17 last (p. 355). While accepting as we do this official denial of the allegations made in that paragraph, we are bound, in justice to ourselves, to say that our information was as categorical as the present disclaimer. In the circumstances we can only express our apologies that the position was misrepresented, and are glad to give the fullest publicity to Dr. Saunders' letter recording the facts so far as the staff is concerned.

### CHRISTMAS AND THE HOSPITALS.

The past week has been a strenuous one for hospital workers. The public hears a good deal about the joys of Christmas in the wards of our voluntary hospitals; what it does not hear about is the careful preparation, the willing personal service, and the energetic activity of hospital workers which go so far to make ward entertainments a success. It is true that in many cases generous outside support is given, but in the majority it is the staff, the nurses and the actual workers who take a pleasure in affording a joyous Christmas to the inmates of the wards. The bill for these entertainments is not a small one, and yet it is always defrayed from the funds specially provided by the staff and the friends who specially know the Christmas needs of the hospitals, and who make a point of ear-marking certain contributions "for Christmas festivities." We should like to see the outside public take a greater share in these entertainments and festivities than it does at present. Let it be remembered that the staff and the regular workers have laboured the whole year through, and that they need as much relaxation at Christmas time as their charges do. There are many subscribers to hospitals who are able, and who would be willing did they only realise that their services would be cordially appreciated, to do their share in contributing to the ward entertainments. At present the public does not know what it can do in the way of personal service to help the hospitals. It should be the duty of hospital secretaries to call attention to the various ways in which such personal service can be made effective, and the manner in which it would be welcomed. At no period of the year is an active, personal interest in our hospitals so beneficial as at Christmas.

### CHRISTMAS SOUVENIRS.

Several hospitals issue Christmas souvenirs, and this annual reminder of the hospital work is valuable to the institution and welcome to the recipients if, as is the case with the neat little pamphlet sent out by the Leicester Infirmary, the souvenir is a work of art and contains really interesting reading matter. The Leicester souvenir is the annual report condensed and explained. It is beautifully illustrated by a series of photographs showing the different wards, views of the nurses' home, and phases of institutional life generally, and is bound to be appreciated by those to whom it is sent. Nowadays the custom of forwarding Christmas cards to friends and acquaintances is almost universal, and there is no reason why institutions should not follow it as well as individuals. And no card we know of can be so useful as such a souvenir. Artistically printed and attractively written, it is not a mere ephemeral production, but a splendid advertisement for a deserving institution—an advertisement, be it added, which is both appropriate and timeous. We hope that other hospitals will take the Leicester example to heart.

### THE PAST YEAR.

In the hospital world several incidents that have marked the past twelve months stand out specially for consideration. The active regeneration of the British Hospitals Association, and the success of its first annual conference held at Glasgow in September last, is perhaps the most important event of the year from a hospital point of view. The action taken by the King's Fund in nominating a special committee for the purpose of investigating the out-patient department problem is another important event of the year; while not less important, although it has received perhaps more than its fair share of attention, has been the Poor Law Commissioners' Report, and the discussion which has attended its publication, and which has gone on with unabated vigour during the year just about to close. The progress which has been made in hospital construction, administration, and technique generally, we have from time to time noticed in these columns. It is gratifying to observe that the specialisation of hospital work has made excellent progress during the year. In England we have welcomed the publication of Dr. Mackintosh's book, which is likely to remain a standard authority for some years, and on the Continent we have noticed the establishment of two special journals which are devoted almost entirely to institutional interests. Coincidently with these signs of more general attention to the great problems of what may be styled institutional politics, in this country, on the Continent, and in America, as evidenced by discussions, papers and resolutions at various congresses and conferences, it is worth while to note that an equally lively attention to some outstanding features of hospital questions has been manifested in the Colonies. The annual report of the Inspector of Hospitals in New Zealand, which lies before us, is one of the most interesting reports issued in the Dominion. We hope to deal with it at length in an early issue, and meanwhile content ourselves with the remark that it gives proof—and ample proof—of the active interest which questions now under consideration in this country are exciting at the Antipodes. Equally interesting, though in a slightly different way, has been the instructive discussion on hospital administration which followed the reading of Sir Kendal Franks' paper before the recent South African Medical Congress. These points cannot be dealt with as they deserve in "Week's Work," and we therefore reserve their full consideration for a future occasion, but they serve to point out the main fact—namely, that there has been a gratifying and wholesome awakening of public interest in the general question of hospital work. This is, so far as the Mother Country is concerned, all the more satisfactory, because, as was pointed out in this Journal a fortnight ago ("A Bad Year for the Voluntary Hospitals," The Hospital *Christmas Appeal Number*, December 17), "politics have broken up the golden days of the year."

### THE DEATH LIST.

Hospital workers have had to deplore the loss of many good friends and valuable helpers during the past year. Here it is only necessary to refer to the death of the late King, an event which has robbed the hospitals of this country of one of their warmest and most active supporters. The death roll includes also well-known names, such as those of Messrs. Nixon and Lucas, of men who have been actively associated with the work of metropolitan and provincial voluntary institutions for many years, and whose loss means a severe blow to the several hospitals with whose interests they identified themselves during life. The Dead Hand, it is satisfactory to note, has benefited the hospitals generously during the year, and although it is as yet too early to summarise the financial position of all the institutions, we imagine that when the final summary is made there will be found a marked increase in the total from legacies and bequests to hospitals during the year. This increase from testamentary donations is likely to be in sharp contrast to the decrease from other sources, as pointed out in the article above alluded to. It is, however, a significant sign of the confidence which the rich man feels in the voluntary institutions of this country—a confidence which the general attack on the system underlying these institutions, which has been so actively pursued during the past year, has not materially tended to shake. All these points must be consoling to hospital workers, and there can be no doubt that anyone who glances back and reviews, in imagination, the year's work will be able to turn with reinvigorated courage and renewed hope to the task of tackling the problems that lie before us in the coming year.

### QUESTIONS OF THE DAY.

What these problems are every hospital worker will know. There exists diversity of opinion as to the manner in which they should be grappled with, and such diversity of opinion is, in its very nature, an encouraging sign, for it is a proof of the fact that these questions are being attacked from various points of view. It is high time that the hospital worker, as such, should have the courage to express his opinions, and to make his conclusions known to the public. He stands in a particularly enviable position with regard to the discussion of sociological matters. On the one side he is in close touch with the medical profession, on the other with the community, and it is no exaggeration to say that the *rôle* of interpreter for both sections should be his, at least in all matters that concern the hospitals. And it is every day becoming more apparent that in all sociological questions the experience derived from the work of our voluntary institutions is likely to play an important part. It therefore behoves hospital workers to take a keener and closer interest in these problems, to make their weighed conclusions known, and to render their experience available for purposes of comparison and collation. The British Hospitals Association, we have no doubt, will take a prominent part in representing the hospital profession, and we look to this influential and representative society with confidence for a determined effort to promote the solidarity of workers throughout the Empire. For we would lay stress on the fact that great questions of sociological interest are not merely metropolitan, not even provincial, but in their best sense Imperial, and that the experience gained in the Antipodes is as useful to us in this country as our experience may be profitable to hospital workers in Auckland or Montreal. We have endeavoured in the past to be a medium for discussion and the interchange of opinions between hospital workers. We hope in the coming year considerably to extend our field of action, and to pay more particular attention to those outstanding problems that urgently call for discussion and consideration. In order to do so successfully we need the help and co-operation of all our readers. The help that we have received during the past year makes us confident that we will not have to appeal for such co-operation in vain.

### THIS WEEK'S DRUG MARKET.

Since last week's report appeared business in the drug market has been practically at a standstill owing to the holidays. No price changes of special importance have taken place. The prospects for the New Year are favourable, and there is good reason to believe that after the period of stocktaking is over business will be resumed with increased activity. The general tendency of prices is in an upward direction, and it should not be without profit to follow the course of the markets with attention. Among the drugs which there is reason to believe may become dearer are ergot of rye, glycerine, cod-liver oil, cascara sagrada, morphine, codeine, camphor, and potassium bromide; but it would be unwise to make too definite a forecast.

